# Biomolecular Mechanisms of Autoimmune Diseases and Their Relationship with the Resident Microbiota: Friend or Foe?

**DOI:** 10.3390/pathophysiology29030041

**Published:** 2022-09-01

**Authors:** Skender Topi, Lucrezia Bottalico, Ioannis Alexandros Charitos, Marica Colella, Marina Di Domenico, Raffaele Palmirotta, Luigi Santacroce

**Affiliations:** 1 Department of Clinical Disciplines, University of Elbasan, 3001 Elbasan, Albania; 2 Interdisciplinary Department of Medicine, Section of Microbiology and Virology , School of Medicine, University of Bari, 70124 Bari, Italy; 3 Department of Precision Medicine, University of Campania “Luigi Vanvitelli”, 80138 Naples, Italy; 4 Interdisciplinary Department of Medicine, Section of Sciences and Technologies of Laboratory Medicine, School of Medicine, University of Bari, 70124 Bari, Italy

**Keywords:** microbiome, microbiota, *Prevotella copri*, dysbiosis, autoimmune diseases, molecular mimicry

## Abstract

The use of innovative approaches to elucidate the pathophysiological mechanisms of autoimmune diseases, as well as to further study of the factors which can have either a positive or negative effect on the course of the disease, is essential. In this line, the development of new molecular techniques and the creation of the Human Genome Program have allowed access to many more solutions to the difficulties that exist in the identification and characterization of the microbiome, as well as changes due to various factors. Such innovative technologies can rekindle older hypotheses, such as molecular mimicry, allowing us to move from hypothesis to theory and from correlation to causality, particularly regarding autoimmune diseases and dysbiosis of the microbiota. For example, *Prevotella copri* appears to have a strong association with rheumatoid arthritis; it is expected that this will be confirmed by several scientists, which, in turn, will make it possible to identify other mechanisms that may contribute to the pathophysiology of the disease. This article seeks to identify new clues regarding similar correlations between autoimmune activity and the human microbiota, particularly in relation to qualitative and quantitative microbial variations therein.

## 1. Introduction 

In 2001, Joshua Lederberg used the term “microbiome” to describe the genetic heritage and environmental interactions of all the micro-organisms that populate a given environment. The term “microbiota” therefore refers to the totality of the individual micro-organisms (i.e., bacteria, fungi, archaea, protozoa, and viruses) that live in and colonize a specific environment at a given time. These microbes form an entire community which may accompany an organism through a symbiotic or potentially pathogenic relationship [1,2]. These micro-organisms participate (both directly and indirectly) in the creation of homeostasis in the human body, consequently affecting our health. The consequences of this can be identified as a generic alteration in the composition of the physiological microbial communities, called dysbiosis. Such an imbalance, i.e., the loss of a mutually beneficial balance between our cells and the micro-organisms that coexist with us, may be associated with a number of diseases [1,3]. In this context, autoimmune diseases play a special role, as the micro-organisms that coexist with us regulate (and, in turn, are tightly regulated by) the immune system. Of the whole microbiome, the intestine and the oral cavity microbiota have a special place, due to the number and variety of micro-organisms therein and their long-known association with autoimmune diseases such as rheumatoid arthritis [4]. 

Autoimmune diseases and increases in their occurrence, both in developed and developing countries, are particularly interesting for the scientific community, highlighting the relationship between the hygiene hypothesis (proposed in 1958 by Strachan) and the microbiota, which can contribute, through various mechanisms, to the development of autoimmunity in the body [5]. Thus, some micro-organisms have co-evolved with our body, forming a beneficial relationship, and their loss may lead to a lack of protection from several autoimmune diseases. The main factors responsible for the establishment of autoimmunity are human HLA antigens, the process of citrullination, and the phenomenon of molecular mimicry. Molecular mimicry is a natural mechanism that underlies a variety of other phenomena; it has been proposed as a means by which to explain the establishment of autoimmunity [6,7]. This phenomenon seems to have been fully studied in rheumatoid arthritis, providing data on the molecules causing autoimmunity and contributing to the elucidation of the pathophysiology of the disease [8]. As a matter of fact, considering the role of the microbiota in the host organism’s health, it is logical to continue studying factors, such as nutrition, which play a dominant role in its formation. Food ingredients, probiotics, prebiotics, as well as whole dietary patterns seem to affect the microbiota and contribute to the autoimmune phenomenon. The development of the concept of the microbiota has given new impetus to the study of nutrition, as these micro-organisms may be associated with the increased incidence of certain diseases [9]. In this regard, autoimmune diseases are the most common; their incidence has been constantly increasing, leading to high monetary and person-hour costs; in addition, such diseases drastically reduce the quality of life of affected individuals. Thus, in light of this new research direction, the study of nutrition and its correlation with human health has become particularly useful and necessary [10]. 

## 2. The Human Microbiota and Homeostasis 

### 2.1. Gut and Oral Microbiota

The micro-organism components of the human microbiota are located on both internal and external surfaces, but most are found in the gastrointestinal tract and in the large intestine, with modern calculations estimating the population size to be 3.8 × 10^13^ bacteria [11,12,13]. 

The extent and composition of the microbiota depend on several factors, which are a key research area in modern science. These factors include the architecture of the organs and their evolutionary history, the host genotype, the immune system, body mass index, diet, lifestyle, and age, as well as treatments (e.g., antibiotics). Phylogenetically related organisms tend to have more similarities in their microbiota than distant species. However, endogenous factors—and specifically in humans—such as general lifestyle and diet are decisive in the composition of the microbiota and, therefore, in the health of the organism. Observing the correlations between micro-organisms and human health, scientists have been prompted to introduce concepts such as dysbiosis and altered crosstalk axes (e.g., gut/brain, gut/liver, gut/skin) [12,14]. 

The largest microbial community is the gut microbiota, which is an “organ factory ecosystem” of trillions of micro-organisms composed of different phyla of bacteria, archaea, fungi, viruses (phages), and eukaryotic micro-organisms that have come to live in the lumen and mucosal surfaces in a co-operative relationship with the human body during its evolution [15,16]. The intestinal microbiota—with one hundred times more genes than the host—evolves over time, adapting to changing living conditions. In fact, the number of communities of microbes increases (both quantitatively and qualitatively) from the proximal to the distal part of the digestive tract. More than 70% of these bacteria cannot be cultivated with conventional methods [17,18]; however, in post-genomic research, the use of advanced molecular techniques (e.g., electrophoresis, immunofluorescence), proteomics, and metabolic analyses has shed light on the composition and function of the human microbiota and the interactions between microbes, the host, and other apparatuses/districts of the organism, as well as the factors that influence them. Next-Generation Sequencing (NGS) techniques make it possible to sequence microbial genomes, the transcriptome, and the epigenome. The 16S amplicon metagenomic sequencing technique is an ultra-deep DNA sequencing method designed to sequence the target genes of 16S ribosomal RNA (rRNA) using universal primers in order to describe and compare the phylogeny and taxonomy of various bacteria (including archaea) and fungi [19,20]. The composition and diversity of the gut microbiota is a result of initial natural selection based on genetic factors, gender, and mode of delivery (natural or caesarean), as well as the intestinal tract, which tends to affect colonization behavior, and other factors. Subsequently, there are numerous affecting factors, including environment, hygiene, diet (e.g., short-chain carbohydrates not being sufficiently absorbed), exercise, stress, drugs (mainly antibiotics), chemical compounds (e.g., bisphenol A), tobacco use, and substance abuse (e.g., alcohol, methamphetamine) [20,21]. 

**Figure 1:**
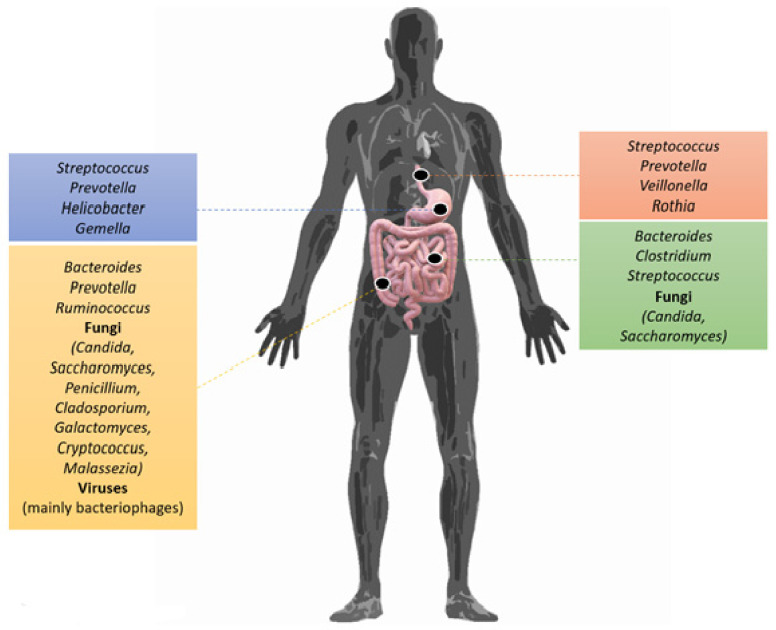
The gastrointestinal tract has a variety of favorable or less favorable habitats for micro-organisms, extending from the oral cavity to the anus. Furthermore, differences in conditions, due to the physiology or the substrates present (e.g., those deriving from food), can act as microbial selection “forces” for each environment. Along the gastrointestinal tract, micro-organisms are predominantly found in the large intestine and ileum, while a smaller number are observed in the jejunum. The stomach and duodenum are colonized by unique species [36,37]. Despite various difficulties, studies have shown that the human gastrointestinal system is dominated by two phyla: *Bacillota* and *Bacteroidota* [38]. Credit: Original figure by I.A. Charitos.

Most of the micro-organisms in the gut microbiota are anaerobic and appear to form a stable “matrix” (including more than 95% of its total composition) in humans, including four dominant gut microbiota phyla: *Bacillota*, *Bacteroidota*, *Pseudomonadota,* and *Actinomycetota*. Meanwhile, the phyla *Pseudomonadota*, *Verrucomicrobia*, *Actinomycetota*, *Fusobacteria,* and *Cyanobacteria* colonize the intestine in smaller quantities. According to one theory, there are three main dominant enterotypes (*Bacteroidota*, *Prevotella,* and *Ruminococcus*), based on the composition of the microbiota and their predominance, which are determined by dietary factors [22,23]. The creation, development, and maintenance of the gut microbiota is a complex process that begins immediately after the organism is born. The types of micro-organisms that colonize the intestinal tract of newborns—which, according to other studies, is sterile—are determined by the mode of delivery, as mentioned above. It has been found that those born via natural birth acquire their intestinal microbiota through vaginal genera such as *Lactobacillus, Prevotella, Atopobium,* and/or *Sneathia*, while those born by caesarean section tend to have elevated levels of bacteria from the skin, such as those of genera *Staphylococcus*, *Corynebacterium,* and *Propionibacterium* [24,25]. Subsequently, the intestinal microbiota develops differently, although this is further influenced by differences in the baby’s diet (breastfeeding vs. breast milk substitutes) and the environment it inhabits. Oligosaccharides in breast milk promote the growth of *Lactobacillus* spp. and *Bifidobacterium* spp., which strengthen the immune system, thus preventing conditions such as eczema and asthma [26,27]. The introduction of solid food leads to an explosion of bacterial diversity in the intestine, with a consequent decline in the populations of *Lactobacillus* spp. and *Bifidobacterium* spp., while bacteria of phylum *Bacteroidota* predominate in neonates [28]. Thus, in the first year of life, the intestinal microbiota may vary dramatically from individual to individual. At the end of the first year, it stabilizes and shows no significant changes compared to an adult microbiome after 20 years. In the microbiota of the elderly, the most notable feature is the altered proportions of phyla *Bacillota* and *Bacteroidota*, with the elderly having a higher percentage of *Bacteroidota*, while young adults have a higher rate of *Bacillota.* Heterogeneity in terms of microbial species can be observed throughout the digestive system; bacterial density gradually increases along the gut, with the colon showing the highest concentration and variety of bacteria [29,30]. Some studies have shown that samples from the small intestine were rich in *Actinomycetota* and *Bacillota*, while those from the colon were rich in *Bacteroidota,* as well as the family *Lachnospiraceae* of the *Bacillota* phylum (Figure 1). Finally, the surface of the intestine is composed of absorbent enterocytes, enteroendocrine secretory cells, and cup-shaped cells that produce mucus. In the immediately underlying chorion, various immune system cell populations can be found, such as neutrophils, macrophages, and dendritic cells, as well as B and T lymphocytes. Direct contact of bacteria with enterocytes is prevented by a protective layer of mucus which, in the small intestine, is easily transported; meanwhile, in the large intestine, this layer attaches and is made up of a protein called mucin. Although this mucus plays a protective role, it also contributes to the bio-coating of bacteria such as *Escherichia coli*, as well as being a source of carbohydrates for bacterial colonies [29,31].

The oral cavity contains the second-largest microbiome in the gastrointestinal tract. The human oral microbiome database has revealed 772 prokaryotic species, 30% of which are currently not culturable. Using 16s rDNA, these bacteria have been classified into six genera and compared with the *Bacillota* phylum [31,32]. The oral cavity microbiota and other micro-organism resident communities in the human body co-evolve simultaneously. Some micro-organisms are first established through the use of various glycoproteins and polysaccharides to create a biolayer (microbial colonization), while appear subsequent to this process. The roles of the oral microbial community in health are many and varied, including energy production and digestion, skin and mucosal preservation, as well as the treatment and detoxification of environmental chemicals [33,34]. Indeed, it can, for example, transform sugar; however, exposure to various agents, such as disinfectants, antibiotics, heavy metals, cigarette smoke, alcohol, and drugs (e.g., methamphetamine, cocaine) may have a negative effect on the oral ecosystem, causing disorders such as caries, periodontitis, inflammation of the mucous membranes, and more [35].

### 2.2. The Role of the Intestinal Microbiota in Human Health 

The intestinal microbiota plays a key role in the nutrition, metabolism, and physiological characteristics of the intestine (e.g., proliferation and differentiation of intestinal epithelial cells, pH, function), in the development of the immune system, and in protecting from pathogens [39,40]. Carbohydrate fermentation is a key activity of the gut microbiota, which aims to produce energy and carbon molecules for the large intestine. It is a process that, in terms of time and action in the gut, involves many steps and several enzymes from many different micro-organisms such as *Bacillota* and *Bacteroidota* phyla species, which secrete enzymes (hydrolases) to break down soluble and insoluble polysaccharides [41]. The initial fermentation of carbohydrates involves digestion in the small intestine following the use and cross-distribution of metabolites by the various members of the intestinal microbiota and, therefore, the synthesis of short-chain fatty acids (SCFAs) such as butyric, propionic, and acetic acid, beyond the production of intraluminal gas (CO_2_, CH_4_, H_2_) [42,43,44]. Short-chain fatty acids have been identified as a link between diet, the gut microbiota, and the host’s energy metabolism [42,43].

On the other hand, propionic acid and acetic acid are directed to the liver and peripheral tissues, where they serve as substrates for gluconeogenesis and lipogenesis. These metabolites not only act on the intestinal mucosa, but also diffuse through it, stimulating the intestinal immune system and, therefore, the immune receptors. Studies have shown that G41 and G43 have protein-coupled receptors GPR41 and GPR43, respectively, which are activated by acetic and propionic acid, while butyric acid activates the specific GPR109A receptor, which helps to reduce intestinal inflammation and inhibit mast cell activation and de-granulation [45,46,47]. The strong anti-inflammatory properties of butyric acid, as well as its participation in immunoregulatory and anti-inflammatory processes, determine the healing of mucosal injuries and the stimulation of mucus production in critical events. Furthermore, butyric acid has a remarkable range of other properties, such as maintaining mucosal integrity, as well as suppressing inflammation and carcinogenesis by affecting immunity, gene expression, and epigenetic differentiation [48,49,50]. A substantial endogenous supply of proteins can be derived from epithelial exfoliation, secretions, and mucus, and the intestinal microbiota plays a significant role in the recycling of body nitrogen and amino acids [51,52]. The synthesis of vitamin K and various components of vitamin B is another important metabolic function of the intestinal microbiota. The microbiota also can metabolize and re-absorb cholesterol and bile acids. Bile acids are biosynthesized in the liver, in peripheral cells, from the precursor molecule cholesterol. The primary bile acids which are produced in the human body, such as the chenodeoxycholic acid, are coupled to taurine or glycine, and are responsible for the absorption of fats and fat-soluble vitamins throughout the small intestine [53,54]. At the level of the final ileum, 95% of these acids are re-absorbed and transported to the liver via the portal vein. The continuous recycling of bile acids, mediated by the enterohepatic circulation, is an important function of digestion [55,56]. The intestinal microbiota participates in the breakdown of various phenolic compounds which are consumed in the diet. Polyphenols are a diverse class of plant secondary metabolites, often associated with the colour, taste, and defence mechanisms of fruit and vegetables. They exist as glycosylated derivatives linked to sugars such as glucose, galactose, rhamnose, ribulose, arabinopyranose, and arabinofuranose [57].

They usually remain inactive in the diet and bio-transform into active compounds after removal of half of the sugar from the gut microbiota. The effect of microbes on drug metabolism is mediated by the enzymatic catalysis of diverse types of reactions, such as reduction and hydrolysis, which can lead to the activation or deactivation of drug components [58]. The most-studied case of enzymatic modification of a drug is that of digoxin, which is used in conditions of heart failure and arrhythmia. According to in vitro experiments, the bacterium *Eggerthella lenta* (previously known as *Eubacterium lentum*) is responsible for the reduction of digoxin to dihydrodigoxin. In particular, the CGR1 and CGR2 genes of the CGR operon encode proteins that are oxidized and use digoxin as an electron acceptor, reducing digoxin to dihydrodigoxin [59,60].

## 3. Microbiota and Biomolecular Processes of Immunomodulation 

**Figure 2:**
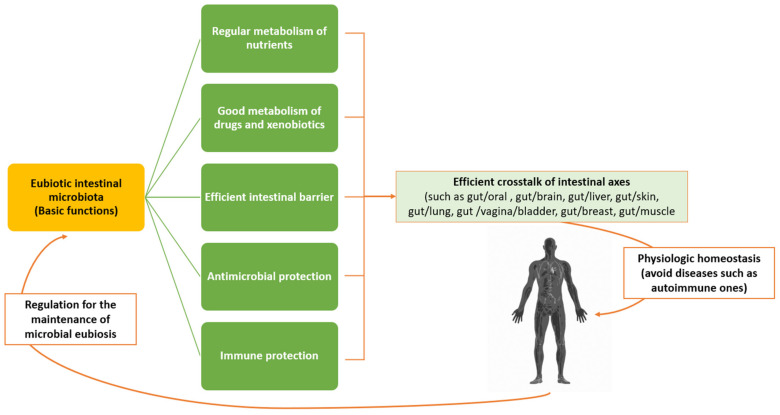
There exists a correlation and a crosstalk-cycle immunity regulation (CCII) between the intestinal microbiota and the various organs, thus building immune network communication pathways under the control of bacteria. In the case of eubiosis, the axes—through correct function of the friendly microbial matrix in the intestine—maintain immune homeostasis and, thus, avoid some diseases. According to the current view, a new cellular balance is considered critical for immune homeostasis: that between Tregs and their functional cells and the different Th subsets. A change in the gut microbiota (i.e., dysbiosis) can disturb this balance, resulting in de-regulation of the immune response and promotion of a variety of disease outcome. Tregs production, indeed, seems to depend on the interaction between the intestine microbiota and the immune system. This emphasizes the basic idea that different microbes lead to the differentiation of immature T cells into different subtypes. Thus, changes in the microbial components of intestinal microbiota, such as those that can occur with exposure to antibiotics, are likely to play a leading role in the case of metabolic disorders, such as obesity, metabolic syndrome, diabetes, and immune disorders [17,65,66]. Credit: Original figure by I.A. Charitos.

Defining a eubiotic microbiota—especially the intestinal one—is difficult, as it is not only colonized by a wide variety of micro-organisms that have dynamic relationships with each other and with the human immune system, but is also strongly affected by diet, which varies from one individual to another and can have short- and/or long-term impacts. However, a key point in the characterization of a eubiotic intestinal microbiota is that it must not predispose the host to a disease, according to a theory which distinguishes three specific enterotypes for each bacterial genus of *Bacteroides*, *Prevotella,* and *Ruminococcus*, which prevails at present [61]. The *Prevotella* enterotype predominates when there is a higher fiber intake, while the *Bacteroides* enterotype predominates under Western-style diets rich in protein and fat. This classification, in addition to facilitating further microbiota studies, has indicated that these enterotypes are extremely stable over time, such that short-term attempts to modify them may fail. The role of the microbiota in the gut is now quite clear; it encompasses a wide range of functions, including the breakdown of carbohydrates, the production of short-chain fatty acids, the production of amino acids, the production of vitamins B and K, and the breakdown of xenobiotics, as well as the angiogenesis of the intestine and the regulation of adipose tissue [62]. However, two other roles are particularly interesting in rheumatic diseases: protection against the establishment of pathogenic micro-organisms and, most importantly, the maturation of the immune system [63,64]. In healthy people, the living microcosm in the intestine (wall/lumen) is in constant dialogue with the immune system, which aims to maintain the homeostasis of the organism. This homeostasis is expressed by consolidating immune tolerance against one’s antigens and ensuring the effective identification and destruction of harmful foreign antigens [65]. Disruption of this delicate balance can lead to infection, inflammation, and cancer. The immune system constantly controls the micro-organisms of the microbiota by blocking their overgrowth through several intrinsic mechanisms such as the production of mucus by goblet cells, the production of antimicrobial peptides by Paneth cells, and the production of IgA by B lymphocytes (Figure 2) [65,66].

Under certain conditions, such as a Western-style diet or the use of antibiotics, the normal microbiota can become “pathogenic” for the host due to changes in the individual composition and proportion of ingredients (dysbiosis) as opposed to the existence of pathogenic micro-organisms. The term “immunity” also includes the concept of the minimal inflammation which is necessary for the normal functioning of the host and flora, and which is ensured by the recognition of a multitude of signals emitted by the microbes comprising the microbiota. These signals include microbial molecules such as peptides, liposaccharides, DNA fragments, peptidoglycans, and the so-called pathogen associated molecular patterns (or PAMPs), which are perceived by specific receptors or host structures. If the germs manage to cross the epithelial barrier, they are confronted with the second line of defense, that is, the phagocytosis of tissue macrophages and the autophagy pathway of intracellular microbes. Activation of the autophagy pathway contributes to defense by influencing the function of Paneth cells, macrophages (M)-φ production, and selection of T-reg cell populations. Furthermore, the production of IgA by B lymphocytes is promoted [67]. The eubiotic microbiota contributes to the formation and integrity of the intestinal barrier function, which prevents the entry of pathogenic microbes or harmful substances into the body. Different specific bacteria and bacterial products capable of inducing Treg cells (e.g., species *Bacteroides fragilis* and *Clostridium* belonging to phylogenetic groups IV and XIV) promote T cell differentiation into Tregs in mice. To the contrary, Segmented Filamentous Bacteria (SFB) promote the differentiation of pre-inflammatory Th17 cells [68]. Through its constant interaction with the immune system (both innate/non-specific and acquired/specific immunity) the microbiota contributes to its maturation, to the effective defense against pathogens, and to the maintenance of the organism’s homeostasis. The main function of the innate immune system is defense and, thus, protection of the host from various diseases. It recognizes a large array of microbial markers, thus activating a series of inflammatory and antimicrobial pathways. Part of this complex system involves interferons (IFNs), especially the IFN-1 family (IFN-α or IFN-β), which can promote the activation of adaptive immunity. Type-1 interferons not only function as signalling molecules for innate immunity, but also promote the activation of adaptive immunity [69]. IFN-1 helps to regulate the growth and renewal of intestinal epithelial cells, thus regulating their apoptosis through promoting the correct intestinal barrier function. IFN-1 also maintains intestinal homeostasis by influencing its microbiota. In fact, systemic IFN-1 can modulate the differentiation of CD4+ T lymphocytes and, subsequently, their function, mediated through its influence on dendritic cells. On the other hand, it has been noted that a reduced abundance of bacteria producing butyrate and acetate is associated with the presence of anti-interferon gamma (IFN-γ) antibodies. Indeed, in inflammatory bowel diseases (IBDs), it has been observed that IFN-γ and over-expressed pro-inflammatory cytokines alter the vascular endothelial barrier in the intestinal mucosa, due to the impairment of the adherent VE-Cadherin protein aggravating its permeability, thus promoting bacterial translocation and worsening the course of the disease [70].

The microbiota can influence the migration and function of neutrophils, as well as the differentiation of subgroups of T cells into Th1, Th2, and Th17 cells, inducing inflammation, and regulatory T cells (Tregs), which inhibit inflammatory routes. Thus, the interactions of the intestinal microbial population with the host play a role in inducing immunity, suppressing inflammation, and maintaining an effective defense against pathogens (e.g., through induction of inflammation or destruction of microbes) [71]. Disturbances at any level of these mechanisms lead to infection or the perpetuation of inflammation; for example, causing the onset of colorectal cancer [13]. Diversity, stability, resilience, and adaptability are characteristics of the microbial community, and the loss of one or more of these characteristics due to certain conditions (e.g., nutrition, immune system, inflammation, infection) is the first step towards the emergence of dysbiosis, which can be harmful. It should be specified the condition of dysbiosis refers to parts of the microbiota that have undergone changes in their composition and functionality due to pressures related to their environment or host, and not to individual or opportunistic micro-organisms [72]. The form in which the phenomenon is expressed is not singular, but may include the development of some pathogenic micro-organisms, the loss of “good” symbiotic organisms, and/or a loss of diversity. Among the factors mutually influencing the microbiota and the immune system, which relates to an enormous antigenic charge, we may consider the micro-organisms and various foods that pass through the gastrointestinal tract [73]. The immune system plays a key role in the body, keeping the microbiota under control through both innate and adaptive immunity. However, this relationship goes two ways, as the immune system must be regulated to ensure the appropriate microbial conditions in order to be effective, and to be able to distinguish the beneficial micro-organisms from the non-beneficial ones. It is now considered that, in light of biological research, various modern diseases are the product of the different speeds of evolution of the genome and the modern environment. For example, genes that once had an evolutionary advantage, such as the insulin gene, are currently an evolutionary burden, causing the onset of diabetes [74]. Changes in symbiotic and dysbiotic interactions appear to be strongly associated with various autoimmune diseases, due to various mechanisms. Sjogren’s syndrome, Crohn’s syndrome, systemic lupus erythematosus, and rheumatoid arthritis are some of the diseases in which there exists a clear association with the oral microbiota, as well as various data indicating the mechanisms of causation. To date, various organizations are candidates for many diseases. It is particularly interesting, however, that *Porphyromonas gingivalis*—the leading cause of periodontitis—is often associated with rheumatoid arthritis. This association has been shown in terms of the double risk of rheumatoid arthritis in patients with periodontitis, as well as by the association of periodontitis with DAS28 (Disease Activity Score for R.A.) [74,75].

## 4. Relationship between the Microbiota and Autoimmunity 

The idea of a functional bridge between the microbiota and certain diseases has been observed from different angles in various body districts; however, these relationships do not necessarily indicate causation. Autoimmune diseases represent a significant problem in the population, as they comprise a heterogeneous group of conditions in terms of prevalence, pathogenicity, clinical scenarios and, finally, the cost of pharmacological treatment [41]. An association exists between the microbiota environment and its effect on the onset of autoimmune diseases. There are three main reasons why an abnormal immune tolerance and/or autoimmune reaction can be induced by dysbiosis. The negative effects of dysbiosis on intestinal mucosal permeability have been noted in patients with autoimmune diseases, indicating signs of their weakening. These weakened intestinal barriers can result in immune exposure to common resident gut bacteria [76]. Furthermore, the disruption of mucosal immune tolerance leads to aberrant and pathological immune responses to the gut microbiota, contributing to the severity of the disease. These include molecular mimicry, bystander activation, and persistent infection with or without local microbial spread (i.e., epitope spread). Molecular or antigenic mimicry pre-supposes a common immunological epitope between a microbe and the host for cross-immune reactions. In autoimmunity and autoimmune disease, nucleic acids and their binding proteins are included in the repertoire of autoantigens [70,77]. Why and how ribonucleoproteins (RNPs) drive this process is still unclear, but their prevalence could be partly explained by their ability to act as ligands for toll-like receptors (TLRs), which are part of the immune system. These receptors act as sensors for endosomal nucleic acids. Thus, TLR recognition of nucleic acid patterns may serve as the signal that activates B lymphocytes. However, in this process, it is possible that autoimmunity and some of these ribonucleoprotein particles may originate from molecular mimicry at a microbial ortholog level. One of the best examples of molecular mimicry in individuals as a mechanism responsible for the development of autoimmune disease is that associated with rheumatic fever, following infections with group-A β-hemolytic *Streptococci* [70,77,78]. Epitope spread refers to autoimmune responses to autoepitopes, occurring when the autoantigen is released in an inflammatory response. These autoantigens can be the result of slightly mutated antigens (even at the level of an amino acid residue). Consequently, the immune response is not specific and will affect both the mutated and non-mutated proteins. For example, a persistent viral infection can cause immune-mediated pathologies, due to the continuous presence of viral antigens that activate the immune system [70,79]. Furthermore, the activation/apoptosis mechanism of adjacent non-infected cells can lead to autoimmune diseases. Indeed, viral infections activate certain antigen-presenting cells (APCs) which, in turn, activate susceptible self-reactive T lymphocytes to trigger an autoimmune response and, hence, the disease. This can spread to uninfected neighboring cells, and their involvement can lead to apoptosis, revealing cryptic self-epitopes which facilitate autoimmune mechanisms. Studies in animal models and humans have indicated the involvement of the gut microbiota in autoimmunity. Thus, the loss of immune tolerance to autoantigens may be the result of dysbiosis, and vice versa [80]. The microbial populations participate, to some extent, in the development and perpetuation of self-reactive immune responses which lead to tissue destruction and the onset of autoimmune diseases, such as autoimmune arthritis—a reactive arthritis (also called Reiter’s syndrome or Fiessinger–Leroy syndrome) caused by bacteria (which are also present in asymptomatic people) that can lead to infections [70,81] by bacteria such as *Helicobacter pylori, E. coli, Salmonella, Clostridioides difficile, Shigella, Campylobacter, Yersinia, Chlamydia trachomatis, Chlamydophila pneumoniae,* and *Ureaplasma ueralyticum* [82]. Genito-urinary infections are favored by dysbiosis of the local microbiota, such as that of the vagina, which can lead to an increase in the population of common opportunistic pathogens, such as those of the genera *Ureaplasma* (e.g., *U. urealyticum* and *U. parvum*) and *Mycoplasma hominis*, leading to bacterial vaginosis, infertility, and adverse pregnancy outcomes [83]. Furthermore, the relationship between periodontitis and rheumatoid arthritis has long been described. However, in recent years, the most notable association has been that of *Prevotella copri* (an anaerobic, Gram-negative bacteria) producing the molecule trimethylamine-N-oxide (TMAO), leading to the development of rheumatoid arthritis. *Prevotella* species are abundant in the periodontal areas, the intestine, and respiratory tract, and their increased presence is considered a risk factor not only for the development of rheumatoid arthritis, but also for cardiovascular events [84].

Innate immunity includes cells and molecules that are not specific against a pathogen and that exist prior to infection. These components are the first line of defense and possess a special ability to recognize patterns (pattern recognition) that are not observed in multi-cellular organisms. The loss of this ability in experimental animals disrupted the defense against micro-organisms, with consequences such as inflammatory reactions or the appearance of diseases. Inflammatory bodies include the polyprotein complexes required by caspase-1 protease to activate various cytokines [75,85,86]. This procedure should be carried out under close supervision, in order to avoid excessive inflammation in the host. However, data have shown that the protozoan *Trichomonas musculis* also activates them to release interleukin (IL-18), having a key effect on Th1 and Th17 cell populations [87,88]. Also of interest are extracellular neutrophil traps (NETs), which are tissue-like DNA structures that can trap and kill micro-organisms. These traps are influenced by the microbiota, where organisms such as *Lactobacillus* spp. reduce their ability to form, while others such as *E. coli* promote NET activation [89]. In the past, autoimmune diseases such as rheumatoid arthritis and systemic lupus erythematosus have been associated with the ability of neutrophils to produce extracellular traps, and the effects of dysbiosis should be clarified in this context. The microbiota also affects adaptive immunity. As mentioned above, some intestinal bacteria (e.g., *Clostridium* spp.) promote the differentiation of T lymphocytes through a growth factor (TGF-β), leading to the formation of Tregs, thus reducing inflammation [90]. Furthermore, intestinal epithelial cells express many adaptive immune system receptors. The constant supply of signals from these receptors is essential for the existence of homeostasis between the microbiota and the immune response. The loss or disruption of this homeostasis leads to disruption of the partition between micro-organisms and the mucous layer, leading to inflammation of the tissue. Furthermore, the microbiota can cause changes through epigenetic modifications [91]. Intestinal bacteria can act by methylating the gene encoding the TLR4 liposaccharide. In this way, various micro-organisms may be able to avoid the tolerance of the immune system by epigenetic suppression of the pattern recognition receptors [92]. A final mode of influence involves the production of metabolites by bacteria capable of signalling changes in translation, either at their site of production or, even, in remote organs. One example is the production of short-chain fatty acids (SCFAs) through the degradation of fibers, which affect histone modification as well as the activation of G protein-coupled receptors [93,94]. In acquired (or specific) immunity, the immune system must be able to present the characteristics of antigenic specificity, diversity, immunological memory, and recognition of self- and non-self-molecules [95]. The lymphocytes express membrane receptors and, thanks to the various random gene rearrangements, can acquire the features of T or B cells [96]. The T cell receptor (TCR), which differs from its B cell counterpart, is found on the cell membrane. Furthermore, they are not specific for a single antigen, but only for antigens combined with a major histocompatibility complex (MHC); a property, called auto-MCH restriction, that is another key distinction from B lymphocytes. During their maturation, T and B lymphocytes undergo two processes of selection: Positive and negative. Positive selection allows only those cells that can recognize MHC self to survive while, in negative selection, cells with a higher affinity for the MHC self-molecules are destroyed, providing cells with self-tolerance [97]. Not all cells die, and some autoreactive T-lymphocytes escape even in healthy individuals, as their action is regulated through two immune mechanisms: Clonal unemployment and clonal suppression. If this regulation is disturbed and the lymphocytes are activated, both humoral and cell-mediated immunity can cause serious diseases. The term molecular mimicry describes the similarity of the antigens of infectious agents to human ones [98]; hence, for example, parasites may avoid the immune system by presenting common antigenic structures, thus inducing tolerance. This aspect can coexist with the phenomena of molecular imitation and cross-reactivity. Molecular mimicry is not a rare phenomenon in nature (Table 1). In 1962, Kaplan at al. reported cross-reactivity in patients with rheumatic fever. As mentioned above, the cause for this was the autoimmune reaction to *Streptococcus pyogenes,* a β-hemolytic *Streptococcus*, due to the homology of the structures on the membrane surface of these bacteria and muscles [99]. The disease often affects children and is particularly concerning due to its effects on the heart. In 1985, the same mechanism was proposed in a study of multiple sclerosis (MS33). An example of this mechanism for inducing an autoimmune response is rabies virus encephalitis; in fact, in the past, rabies brain cell cultures have been used to develop a rabies vaccine, despite the fact that they contain rabbit brain cell antigens. These antigens could trigger the patient’s immune response by producing antibodies and activated T cells, which led to encephalitis. Subsequently, the same mechanism after vaccination with the influenza virus H1N1 was noted, leading to an increased risk of developing GBS (Guillain–Barre syndrome) [99,100,101].

**Figure 3:**
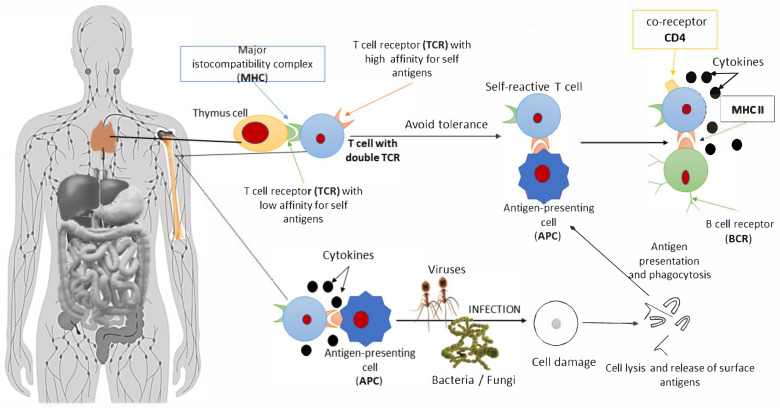
The proposed mechanism of immune system avoidance through tolerance due to the phenomenon of molecular mimicry. T cells with different α and β chain arrangements and dual TCR receptors bind either microbial and self-antigens (presented by antigen-presenting cells), leading to the production of cytokines and autoantibodies [73]. There are four types of molecular mimicry: (a) Absolute identification of the protein presented by the host; (b) homology at the protein level; (c) common sequences of amino acids or epitopes between the host and the micro-organism or environmental factors; and (d) structural components of the micro-organism or environmental agent. Molecular mimicry, considering evolution, is another strategy by which organisms increase their chances of survival. The concept of molecular mimicry must include four criteria to be valid: (a) Similarity between an epitope of an organism and an epitope of a micro-organism or environmental factor; (b) patients with autoimmunity in whom a cross-reaction is observed; (c) epidemiological link with exposure to the respective environmental factor or micro-organism; and (d) reproducible autoimmunity in animal experimental models [73,99]. Credit: Original figure by I.A. Charitos.

Molecular mimicry is a result of the similarity of the myelin sheath in the human body with the structures of viruses, and data have been presented supporting possible correlations with vaccine adjuvants. Due to the low incidence of this side-effect, genetic pre-disposition is the most likely explanation [102,103,104]. MHC class I, II, and III genes encode various useful proteins in different cells. The chains of MHC II molecules are encoded by the DR, DP, and DQ regions, and some of these alleles show greater affinity in people suffering from certain diseases, including various autoimmune diseases. Another example is the relative risk calculation, which is carried out by dividing the frequency of the HLA allele in the patient population by the frequency of the HLA allele in the general population [105], which allows us to correlate genes with their respective diseases. For example, people with the B27 allele have a relative risk of 90, being 90 times more likely to develop ankylosing spondylitis than the general population, while people with the DR4 allele are 10 times more likely to develop rheumatoid arthritis. However, the existence of an association between a gene and a disease does not imply causation; indeed, the associated relationships are typically overly complex and non-linear [106,107]. Indeed, some researchers believe that such mechanisms cannot stand alone, as most of the population interacting with the same micro-organisms do not develop autoimmunity. Bacterial and viral peptides also show 99.7% homology with the human proteome. Researchers have hypothesized that only homology in structure cannot cause autoimmune diseases but, instead, further processes are also involved. This is the case when considering the association between patients infected with Epstein–Barr virus (EBV) and multiple sclerosis. Those carrying the HLA-DRB1 * 15:0 allele have been shown to be more likely to develop it than those carrying either the virus or this allele alone. Molecular mimicry self-reactivity can be achieved in three ways: (a) via TCR multiplicity; (b) through the presence of double TCR receptors in cells; and/or (c) through the presence of chimeric TCR receptors, in terms of structure (α and β chains) [108]. TCRs have high specificity for the molecule attached to the MHC, as the sequences binding MHC II or MHC I molecules consist of 8–10 and 14–18 amino acids, respectively [109]. However, beyond the point where it binds, other regions of the antigen can also bind, thus conferring some plasticity (polyhedrality) to the TCR. However, 30% of human T cells have a double α chain, while about 1% have more than one β chain. [110]. These cells can escape immune tolerance, the mechanism for which remains unknown. One hypothesis is that, while autoreactive T cells are cleared in the thymus gland, those with a double TCR on the surface may be able to escape [111]. A TCR double receptor T cell could recognize an exogenous antigen from an antigen presenting cell (APC), activating it and attacking the epitopes in the body due to the second receptor (Figure 3) [112].

The correlation between the environment and its effect on the onset of autoimmune diseases is not a new theory; for example, considering the emergence of allergic rhinitis in children who have many siblings. The core idea of the Hygiene hypothesis is that some micro-organisms in the microbiota have co-evolved with our body in a beneficial relationship, and that their loss, due to modern hygiene, may lead to a lack of protection against various autoimmune diseases [113,114]. Data supporting the hygiene hypothesis have come from epidemiological studies, in which populations from areas with a low prevalence of autoimmune diseases migrate to areas with high prevalence. Studies on the protective effect of external agents have revealed a reduced incidence of allergies in children who have participated in agricultural activities, where soil-transmitted helminths, such as *Schistosoma* (genus of trematodes), seem to act in a protective manner against the onset of atopy. More compact data supporting the hygiene hypothesis have been derived from animal studies and some human intervention studies [115,116]. Studies on the incidence of type 1 diabetes in NOD (non-obese diabetic) mice, which very rarely exhibit the disease in conventional facilities, have shown an incidence rate close to 100% in populations developed in an environment free of specific pathogens. The position of the microbiota in hygiene theory is important, considering the various microbial populations that coexist with the host, especially the intestinal ones (which make up most of the organisms which we often come into contact with) [117]. Microbiota studies have already provided some examples related to other diseases strongly associated with micro-organisms, such as idiopathic inflammatory bowel disease (IBD) and Crohn’s disease. Crohn’s patients have shown limited diversity of the *Bacillota* phylum; particularly *Faecalibacterium prautsnitzii*, which appears to play a protective role, based on rodent studies. This protective effect appears to extend into the supernatant culture of *F. prautsnitzii*, indicating the existence of a molecule that it is secreted by the micro-organism which has anti-inflammatory properties [118]. *Bacteroides fragilis* also appears to provide protection, as it protected experimental animals from colitis, for which the responsible molecule is known: Polysaccharide A (PSA) [114].

**Figure 4:**
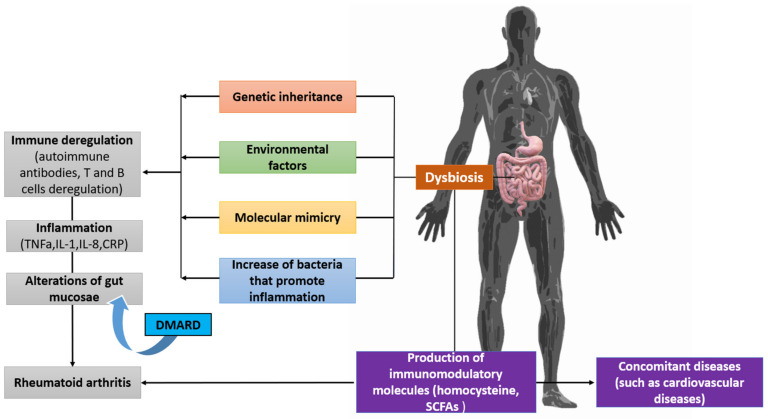
Dysbiosis and its association with the pathophysiology of rheumatoid arthritis. The molecular mimicry phenomenon, as well as the increase in the number of bacteria associated with inflammation, lead to de-regulation of the immune system [127]. Credit: Original figure by I.A. Charitos.

The complete biomechanism of the pathogenesis of rheumatoid arthritis has not yet been revealed; however, it is now clear that both our genome and various environmental factors are involved in the disease, making it more difficult to obtain a clear understanding of its etiology. Starting from genetic factors, the major histocompatibility complex (MHC) is a group of genes on chromosome six in humans, also called the human leukocyte antigen (HLA) complex [119]. The genes at these sites are organized into regions that encode three classes of molecules: MHC I, MHC II, and MHC III. As the MHC genetic loci are located close to each other, most individuals inherit these genes as a set of alleles called haplotypes. MHC II genes are organized into three regions: DP, DQ, and DR [120]. According to the hypothesis of common epitopes, MCH II is responsible for the pathogenesis of autoimmune diseases. In particular, the HLA DRB1*01 and HLADRB1 * 04 antigens contain five amino acids in the antigen presentation area which are closely related to the onset of rheumatoid arthritis. Furthermore, using GWAS technology, some different genetic loci have been found to be associated with the onset of the disease [121,122]. The association between microbiota and rheumatoid arthritis has been observed in many studies. It has been found that a change in the microbiota occurs, which is related to the activity levels of the disease; furthermore, after treatment, the microbiota tends to return to its former composition [123]. An important part in the pathophysiology of rheumatoid arthritis is citrullination, which is an irreversible post-translational modification by the basic enzyme peptidyl-arginine deiminase (PAD). The presence of autoantibodies, which enhance the catalytic activity of PAD4 before its inactivation by the extracellular oxidative environment, further enhances citrullination while a large number of neutrophils are dying in the joint, consistently delivering enzymes to the extracellular environment [124]. The role of the microbiota is already involved at this point, as the bacteria that colonize the body’s mucous membranes also rely on the toxins, which cause pores on the cell surface of neutrophils. This ability puts pressure on them to initiate hyper-citrullination by the bacteria and, after an anti-citrulline protein antibody (ACPA) response is formed, the body itself—thanks to perforin and the membrane attachment complex (MAC)—can maintain the reaction. Beyond its relationship to the citrullination process, the microbiota has been associated with many hypothetical mechanisms in the onset of rheumatoid arthritis. These hypotheses revolve around the role of micro-organisms in the maturation of the immune system, as it is essential both for the mechanisms of de-regulation of tolerance and its relationship with the creation and suppression of inflammation (Figure 4) [125,126].

Under stressful conditions, such as exposure to extreme temperatures, a specific family of proteins can be produced by cells as response. The immune system encounters so-called bacterial heat shock proteins (HSPs), which have the same structure in both prokaryotic and eukaryotic cells. Immunization with these proteins (HSP 60, HSP 70, HSP 40) leads to an increase in anti-inflammatory and a decrease in proinflammatory cytokines, once again linking the role of the immune system in rheumatoid arthritis to the microbiota [128]. Even more immediately observable is the role of the microbiota in the regulation of immune cells with the products of their metabolism; in particular, through butyrates, which have nutritional and anti-inflammatory effects on epithelial cells. Meanwhile, its receptors are downregulated in diseases such as idiopathic inflammatory bowel disease. Butyrate production has been found to contribute to the promotion of Treg differentiation, which otherwise correlates the microbiota with the specific disease [129]; however, its relationship to rheumatoid arthritis is based on the observation that these patients have more Th-17 lymphocytes and fewer Tregs than the healthy population. Th-17 with production of IL-17 is characterized by inflammatory activity while, to the contrary, the Treg population has a key role in maintaining intestinal homeostasis as well as in controlling non-self tolerance [130,131]. A similar observation, with butyrate contributing to the promotion of Treg differentiation, was also made with respect to the polysaccharide A, produced by the species *B. fragilis*, demonstrating that the microbiota has many actions and is not isolated in the organism [132,133]. Moreover, the bacterium *P. copri* appears to play a key role, as it has epitopes in common with HLA DRB1. These common epitopes have been linked to the onset of rheumatoid arthritis, and *P. copri* appears to be more numerous in the early stages of the disease. Colonization of the intestine with *P. copri* increases the risk of rheumatoid arthritis by 1% to 3.95%. It is interesting to note that these bacteria can (and do) metabolize the L-carnitine present in red meat into N-trimethylamine oxide (TMAO), which increases and accelerates atherosclerosis [134,135]. Therefore, such a mechanism could explain why patients with rheumatoid arthritis tend to have increased cardiovascular risk [128].

The genus *Prevotella* is abundant in the human body, without causing infections. The low pathogenicity of these bacteria and their several presences indicate the co-evolution of these micro-organisms with humanity. *Prevotella* genus appears to play a dominant role in the colonization of various mucosal surfaces, is a dominant genus in the respiratory system, and is a major genus in the three enterotypes theory [136,137]. The first association between species of the genus *Prevotella* and diseases, characterized by the presence of chronic inflammation, can be dated to 1928, where it was associated with gingivitis and periodontitis. This genus activates the release of interleukin 1β (IL-1β), 6 (IL-6), and 23 (IL-23) from dendritic cells via the TLR receptor which, in turn, promotes the production of interleukin 17 (IL -17) from Th17 (T helper) cells [138]. The association of *Prevotella* with rheumatoid arthritis has elucidated the mechanism of molecular mimicry as a method for avoiding immune and autoimmune micro-organisms [139]. Suspicions have arisen regarding the association of dysbiosis and the pathogenesis of the disease. Furthermore, activated T-cell mediation has been observed in arthritic mice with intestinal dysbiosis [140]. In addition, it was noted that, in patients with rheumatoid arthritis and dysbiosis microbiota, *P. copri* appeared in greater numbers in the early stages of the disease [140]. A change in the composition of the microbiota has also been observed in patients after the use of DMARDs (Disease-Modifying Anti-Rheumatic Drugs), which correlates the phenomenon of dysbiosis with the clinical picture. Despite the indirect correlations found in the study of dysbiosis, the only known autoantigens that have been shown to be associated with rheumatoid arthritis are ACPAs. [141,142,143]. Using liquid chromatography coupled with mass spectrometry, T cell epitopes have been identified directly from inflamed synovial tissue, then tested for immunogenicity using patient samples. In one study, it has been noted that a peptide from a protein of *P. copri* 27 kDa (Pc-p27) stimulated T and B lymphocytes in 40% of patients with rheumatoid arthritis, but not in the control group or in patients with other rheumatic diseases [8,144,145]. This peptide is also presented by an HLA-DR receptor, which is consistent with existing data on HLA-DR and its relationship to the phenomenon of molecular mimicry. Other studies have identified two other immunogenic peptides presented by the membrane receptor HLA-DR, deriving from the N-molecule acetylglucosamine sulphate (GNS) and phylamine A (FLNA) [146]. GNS is a lysosomal enzyme present in all cells, which is involved in the catabolism of heparin and cartilage components, while FLNA is essential for binding actin molecules in the correct form. These two molecules are expressed in large quantities in synovial fluid, they are targets of T and B cells in patients with rheumatoid arthritis, and sequencing for homology testing between their epitopes and epitopes of microbial origin showed high levels of similarity [147]. Specifically, in the bacterium *P. copri,* the homology was found to be 67% with a periplasmic protein of the bacterium, while FLNA presented 80% homology with an uncharted protein of the micro-organism. In both cases, the maximum homology was to amino acids belonging to the HLA-DR receptor binding region. The corresponding homology was also tested in the micro-organism *P. gingivalis,* without positive results. Interestingly, the immunogenicity of these autoantigens refers to high-affinity IgA antibodies which require T and B cell interaction, not merely low-affinity antibodies whose production is independent of T cells. At the end, the intestinal mucosal barrier breaks down due to dysbiosis or inflammation of the intestine, with the result that micro-organisms may enter and activate the T lymphocytes with cross-reactivity after the activation, due to the molecular mimicry of the autoantigens. Antigen-presenting cells are then involved, increasing the effect. These observed data can help to improve diagnoses, as 69% of patients are positive for the two antigens used for diagnosis (ACPA and RF), while the addition of GNS and FLNA increased this rate to 86%. Additionally, new antibiotic regimens targeting *P. copri* and dietary interventions can be harmonized with the data, in order to obtain improved therapeutic effect [8].

Spondyloarthritis (SA) comprises several related diseases with distinct phenotypes, including ankylosing spondylitis (AS), reactive arthritis (RA), psoriatic arthritis (PSA), arthritis associated with IBD, and a subset of juvenile idiopathic arthritis. Both environmental and genetic factors are responsible for the onset and development of SA. However, the reasons still remain unclear. The presence of the HLA-B27 gene has a direct effect on the development of SA [148]. Among non-genetic factors, it has been observed that the gut microbiota is differentiated in individuals with early-onset SA, compared to that of controls, and an imbalanced gut microbiota likely mediates the activation of inflammatory pathways seen in these diseases. Previous studies have revealed that the proportions of certain bacterial families were increased in the gut of patients with SA, including the *Bacteroidaceae*, *Porphyromonadaceae*, and *Prevotellaceae* families [149]. The effects of HLA-B27 on the gut microbiota and dysbiosis in SA are closely related to the host’s genetic background and/or environment. Histological analysis has shown that both HLA-B27-transgenic Lewis rats and HLA-B27-transgenic Fischer rats developed intestinal inflammation; however, the Lewis rats were resistant to the effects of HLA-B27. HLA-B7-transgenic rats in the control group were not affected. The microbial gut changes in HLA-B27 transgenic rats were strikingly divergent among the three different host genetic backgrounds, including different patterns of dysbiosis in HLA-B27-transgenic Lewis and HLA-B27-transgenic Fischer rat strains, with some overlap. The resistance of Lewis rats to SA may be due to the lack of SFB, which promotes the development of CD4+ Th17 cells [150]. Colonization of specific intestinal bacteria was sufficient to cause a different phenotypic disorder in SA. In contrast, the gut microbiota of patients with SA was characterized by an increased abundance of the phylum *Pseudomonadota*, considering DNA sequencing data based on the 16S rRNA gene and ITS2, possibly due to enrichment of *Escherichia, Shigella*, *Veillonella,* and *Lachnospiraceae* NK4A136, as well as a reduction in *Prevotella* strain 9, genus *Megamonas,* and genus *Fusobacterium.* Moreover, the gut microbial composition of patients with SA presented increased levels of the phylum *Ascomycota* (especially of the class *Dothideomycetes)* and decreasing abundance of the phylum *Basidiomycota*, mainly due to the decline in *Agaricales*. In addition, ITS2/16S biodiversity indices and altered bacterial–fungal interaction networks have been observed in SA patients, compared with healthy controls. Additionally, significant increases in the abundance of *Erwinia* and *Pseudomonas* and an increased prevalence of typical enteropathogens (e.g., *Campylobacter, C. difficile, E. coli*) have been observed in RA patients. [151,152].

Systemic lupus erythematosus (SLE) is a chronic autoimmune disease characterized by persistent inflammation in many organs of the body. SLE mostly affects women and is triggered by complex interactions of different factors (e.g., genetic pre-disposition, hormonal and environmental factors, and so on), which are not entirely clear. Changes in the composition of the microbiota have been hypothesized to be involved in the etiology of SLE, and several studies have been conducted to demonstrate that gut microbiota dysbiosis affects the onset and development of SLE. A decrease in genus *Ruminococcaceae,* an increase in phylum *Pseudomonadota,* a lowering of the *Bacillota*/*Bacteroidota* phyla ratio, and increased abundance of *Bacteroides* spp. in stool samples has been reported in these patients [153,154]. Moreover, in studies of SLE models in mice, gut microbiota compositions have been shown to differ from those of healthy controls. *Lactobacillus* spp. in feces was found to be enriched in a subset of SLE patients and, using a TLR7 knockout mouse model, *Lactobacillus reuteri* seemed to exacerbate autoimmune manifestations, which were inhibited by dietary starch (RS) through SCFAs [155]. Aryl Hydrocarbon Receptor (AhR) ligands involved in the pathogenesis and development of SLE have also been identified. In a study, it has been shown that the level of AhR expression on neutrophils was significantly higher in SLE patients than in healthy subjects, where the proportion of Th17 cells over-expressing AhR was significantly increased in SLE patients, compared with the control group. In the group of patients with high AhR expression, patients with skin lesions, allergies, and lower levels of the C3 fraction in the complement were more frequent than in the group with low expression of AhR, indicating AhR as a potential biomarker for predicting skin lesions in SLE. Indole-3-carbinol (I3C) mediates the activation of AhR, contributing to immunoregulatory effects on macrophages of SLE patients by reducing the expression of pro-inflammatory cytokines and over-expression of anti-inflammatory cytokines [156]. Gut dysbiosis characterizes patients with SLE and, therefore, can be used to diagnose SLE and predict disease activity. SLE patients reportedly exhibit characteristic patterns of gut dysbiosis which are directly related to disease activity. Among the microbes associated with SLE, the genera *Streptococcus, Campylobacter,* and *Veillonella* (e.g., *Streptococcus anginosus* and *Veillonella dispar*) have been found to be closely associated with disease activity, while the genus *Bifidobacterium* showed a negative association. Metabolic pathways differed not only between SLE patients and healthy controls, but also between SLE patients with and without active disease [157,158]. Fecal 16S rRNA analyses have indicated that SLE patients have lower species richness than healthy subjects. It has also been found that patients with SLE presented an overall increase in *Ruminococcus gnavus* (family *Lachnospiraceae)*, which was directly proportional to the overall disease activity, and were more abundant in patients with nephritis [159]. The microbiota indicated an increased presence of gram-negative bacteria, which differed in the genera *Odoribacter* and *Blautia,* as well as an unnamed genus from the *Rikenellaceae* family, and gut microbiota dysbiosis has also been reported in experimental mouse models; however, differences have been found between mouse and human models, including those related to diversity [160]. Another study has compared the levels of antioxidants in serum and gut microbiota with serum levels of malondialdehyde (MDA) and C-reactive protein (CRP) in twenty-one subjects with inactive SLE. In this study, serum copper content was positively associated with CRP levels, and CRP was also positively associated with the proportions of the fecal phyla *Lentisphaerae, Pseudomonadota,* and *Verrucomicrobia*.

Targeting the gut microbiota may be one of the keys for treating SLE. For example, treatment with phosphorylcholine (TPC) and a conjugate of tuftsin improved both immunological and clinical parameters, which was accompanied by a decrease in genus *Akkermansia* and an increase in *Clostridiaceae* and *Mogibacteriaceae* families, as well as *Bifidobacterium*, *Turicibacter, Adlercreutzia*, *Allobaculum,* and *Anaeroplasma* genera correlated with the clinical course and serological parameters of SLE [161,162].

Intestinal inflammation is closely related to chronic joint inflammation. *Prevotella* spp. can be found in various tissues, other than the intestine, spleen, liver, lung, serum, eyes, and joints [84]. More than half of patients have microscopic mild intestinal inflammation (often similar to Crohn’s disease in its early stage), and there is a significant variation in gut microbial composition between SA patients who have this inflammatory condition, compared to those without overt involvement. An increase in *Dialister* genus was found to be positively linked to clinical activity of SA, while a strong reduction of this genus was observed in the non-inflamed ileum and in the biopsy tissues of the colon of patients with SA and healthy controls [163,164].

IBDs can be divided into Crohn’s disease (CD) and ulcerative colitis (UC), which are chronic inflammatory diseases of the gastrointestinal tract resulting from an inappropriate immune response/autoinflammation. Causative factors of IBD include genetic pre-disposition, immune and intestinal responses of the microflora, and environmental stimuli. According to one hypothesis, protein alteration of intestinal extracellular molecules can mediate aberrant host-microbe interactions in IBD [165]. Patients with Crohn’s disease tend to present a reduction in *Bacillota* phylum; specifically, *F. prautsnitzii*. This bacterium appears to play a protective role and have anti-inflammatory properties. *Bacteroides fragilis* also appeared to be protective in experimental animal colitis, mediated by polysaccharide A [166]. In comparison, intraepithelial lymphocytes (IELs) are T cells having closer contact with the intestinal bacteria, which may be influenced by the dissimilarity of microbiotal composition in distinct subtypes of IBDs. IELs and cytokines produced by IELs correlate positively or negatively with the relative abundance of different bacterial families. Compared to healthy humans, IELs from UC subjects secrete significantly greater amounts of IL-1β, and individuals with CD secrete significantly higher amounts of IL-17A, IFN-γ, and TNF-α [167]. Healthy individuals showing a reduction in genus *Roseburia* have a high genetic risk for IBD. Additionally, the disease site is an important determinant of the gut microbiota, with patients with ileal CD showing reduced diversity compared to those with colonic CD. In general, the interaction between the host genome and the gut microbiota based on individual differences can affect health. Increased levels of phyla *Actinomycetota* and *Pseudomonadota,* along with relatively decreased levels of *Bacillota*, have been strongly associated with IBD severity [168,169]. *C. difficile* infection (CDI)—a common complication in IBD—can also cause poor outcomes. IBD patients with *C. difficile* infection had a more specific microbial dysbiosis, with higher levels of genus *Enterococcus* and lower levels of genera *Blautia* and *Dorea* than IBD patients without CDI. In general, it appears that patients with IBD present less diversity and richness in the gut microbiota [170,171]. The immune system of the intestine—particularly, the mesenteric root’s lymph nodes, the resident microbiota, and the liver—acts as a second line of defense to eliminate bacteria that have crossed the intestinal barriers [172].

Hence, the liver is not only a receptor, but also a filter, considering the presence of Kupffer cells, hepatic endothelial and biliary epithelial cells, and pattern recognition receptors (PRRs), all of which are capable of detecting pattern-associated microbial molecules (PAMPs) such as bacterial LPS, peptidoglycans, flagella, and bacterial DNA, among other ligands. An excessive immune response induced by these PAMPs is believed to lead to liver damage, which can evolve into fibrosis [173,174]. Consistent with this hypothesis, it has been hypothesized that mucosal T lymphocytes are abnormally activated by the resident microbiota, causing them to migrate further into the liver; as such, the antigens present in the liver have been investigated. Given the coexistence of IBD and primary sclerosing cholangitis (PSC) in 60–80% of cases, the gut/liver axis has been linked to the pathogenesis of PSC. In general, the gut microbiota shows reduced microbial diversity, when compared to healthy subjects. Changes in the abundance of specific bacteria, such as *Enterococcus* and *Veillonella* genera, have been observed in several studies, which could be treated as biomarkers of PSC [175,176].

Sjögren’s syndrome (SS) is a systemic autoimmune disease that usually affects middle-aged women; it is characterized by lymphocytic infiltration of the exocrine glands, usually resulting in xerostomia and conjunctivitis. Severe intestinal dysbiosis is more evident in patients with primary SS than in healthy persons. Furthermore, SS patients have a higher calprotectin in stools and lower C4 concentrations in blood samples, suggesting a link between intestinal dysbiosis and intestinal inflammation, systemic inflammation, and disease severity [177]. Other studies have also reported associations between the gut microbiota and clinical/laboratory parameters assessing the severity of SS, particularly those associated with dry eye symptoms. The gut microbiota of these patients presents an abundance of *Bacteroides*, expressed as a lower *Bacillota*/*Bacteroidota* phyla ratio. We must keep in mind that certain bacteria can be involved in the development of SS but, on the other hand, decreased saliva production and mouth dryness certainly affect the oral microbiota, thus making the origin of the dysbiosis itself difficult to diagnose [178]. The prevailing opinion—that reduced salivation is the most important factor in the formation of the oral microbiota dysbiosis—appears to be contested, and the abundance of the genera *Lactobacillus*, *Haemophilus,* and *Neisseria* has been significantly correlated with the rate of salivary secretion [179].

A significant contribution of non-genetic factors to the development of type-1 diabetes (DM1) is a marked discrepancy between individuals who carry HLA alleles associated with DM1 and those who do not develop the disease. The destruction of the cells of Langerhans islets occurs when genetically pre-disposed individuals are exposed to certain environmental factors, possibly infectious (i.e., mumps), that activate autoreactive lymphocytes to produce autoantibodies [180]. The intestinal microbiota could be involved in the pathogenesis, i.e., as a link between the intestine and the pancreatic immune system. Initially, studies regarding the relationship between the microbiota and the autoimmune reaction in non-obese diabetic mice (NODs)—who rarely develop the disease—showed an incidence near to 100% in populations from an environment without pathogenic organisms [181].

Indeed, the hypothesis of molecular mimicry by the microbiota in the disruption of immune tolerance to DM1 has been supported by the observation of accelerated DM1 development due to gut microbiota dysbiosis in MyD88 mice [182]. A potential microbial peptide that mimics the islet-specific glucose-6-phosphatase catalytic subunit-related protein (IGRP) has previously been described. Conversely, the expression of some MHC-II alleles may protect NOD mice from autoimmune islet aggression appears to be mediated by the eubiotic microbiota, highlighting the possibility of microbial modulation for the prevention of autoimmune diseases. Furthermore, intestinal microbial metabolites also appear to be protective, and feeding NOD mice with a combined diet facilitating acetate–butyrate production prevents the development of disease, mainly due to acetate-blocked self-reactive T cells and Treg cells enhancement by butyrate [183]. Finally, it has been noted that the transfer of the symbiotic bacterium *A. muciniphila* to NOD mice significantly reduced the incidence of SDI, rather than the entire microbial community, probably due to its multiple effects in the re-modelling of immunological and metabolic signalling [184]. It has been noted that, through the co-metabolism of microbes, the human intestinal microbiota has evolved to exert a significant influence on health and disease, due to the immune interactions of the gut/brain axis.

Recent studies in children with autism have shown increased SCFAs, indicating intestinal dysbiosis, which may affect the expression of genes related to CNS development. Furthermore, they have shown reduced microbial biodiversity accompanied by an increase in some genera of microbes, such as *Clostridium*, *Bifidobacterium*, *Lactobacillus,* and *Bacteroidota,* as well as *Bacillota* phylum [185,186]. Given the wide range of the autism spectrum and the inherent diversity in the gut microbiota of patients, the research evidence is still inconclusive, regarding the causal relationship between the gut microbiota and autistic disorders. In a study on the microbiota and metabolome of children with autism, the authors summarized their observations with pervasive developmental disorder-not otherwise specified [187]. In line with these observations, healthy children with Pervasive Developmental Disorder-Not Otherwise Specified (PDD-NOS) and children with autism presented an altered microbiota and an altered metabolome (including neurotransmitter molecules). It has been hypothesized that the degree of microbial alteration is related to the severity of the autistic disease, as the variations in both the microbiota and the metabolome were greater in children with autism than in healthy children. Finally, it was found that the levels of free amino acids and volatile organic compounds differed in children with autism, compared to children with PDD-NOS, who appeared to have levels similar to those of healthy children [188]. In fact, the studies indicated that bioactive nutrients and the gut microbiota can alter either DNA methylation or the histone signatures through several biomechanisms. Indeed, microbes within the human gut can play an important role in regulating various components of the gut/brain axis through their effect on inflammatory cytokines and the production of antimicrobial peptides, affecting the epigenome through participation in the production of SCFAs, synthesis of vitamins, and absorption of nutrients. In addition, they may be involved in the production of the most common neurotransmitters within the gut. Therapy with probiotics and prebiotics is limited, as only certain bacterial strains can be administered. However, it should be emphasized that studies have indicated the possible beneficial effects of probiotics in the restoration of intestinal dysbiosis and the symptoms of autism [189]. Fecal microbiota transplant (FMT) ensures the transfer of hundreds of bacterial strains simultaneously directly to the colon, bypassing the digestive system. A stool sample is isolated from a healthy donor, which is transferred freshly to the recipient within eight hours (or within eight weeks, if frozen immediately after collection). However, FMT could cause problems, if the donor transfers opportunistic pathogens or silent infections to the recipients [190,191]. Microbial composition and the course of gastrointestinal symptoms in eighteen autistic children after FMT has been evaluated for a period of 7–8 weeks after oral administration of vancomycin and bowel debridement. The results showed that gastrointestinal disturbances (e.g., constipation, diarrhea, or abdominal pain) were reduced by 80% over a period of at least eight weeks. In addition, marked improvement was also observed in seventeen behavioral symptoms of the autism spectrum disorder (assessed through the “Parental Global Impressions-III” questionnaire). This change in the quantitative and qualitative microbiota for some fecal bacteria was demonstrated in terms of the abundance of some of them, such as those of the genera *Bifidobacterium*, *Prevotella,* and *Desulfovibrio* [192]. Therefore, it can be deduced that the gut brain/axis is closely related to a possible lower microbial diversity, which is modulated through metabolites that can modulate not only intestinal symptoms, but also behavioral ones. Hence, FMT provides a very interesting new therapeutic option, which could be used to modulate dysbiosis in patients with autism [193,194].

The onset of the autoimmune reaction in people who have a genetic pre-disposition to Multiple Sclerosis (MS) has been attributed to environmental factors, particularly microbial infections. This is based on data from experimental models of autoimmune encephalomyelitis (EAE), which indicated that the microbiota is required to induce self-reactive anti-myelin B-cells to the autoantigen oligodendrocyte myelin glycoprotein (MOG). On the other hand, oral administration of *Bacteroides fragilis*-derived molecules showed protective effects from demyelination and central nervous system inflammation, induced by tissue-specific expansion of CD4 + Foxp3 + Tregs expressing CD39 in mice [195]. Furthermore, it has been noted that the metabolism of tryptophan into AhR agonists by the gut microbiota appears to be involved in the gut/brain axis, and a reduction in circulating AhR agonists was observed, compared to healthy controls. Many studies have also observed the greater abundance of genera *Akkermansia*, *Butyricimonas,* and *Methanobrevibacter*. [196,197].

## 5. Conclusions

Based on the reported data, the intestinal microbiota can be said to be involved in the pathological mechanisms of various diseases, including IBD, DM1, and autism, among others. The main findings of the studies that have been conducted on the associations between these diseases and microbiota involve the differences in gut microbial composition between patients and healthy individuals. Autoimmune diseases have been associated with gut microbiota dysbiosis and, in some cases, significant correlations have been reported between these diseases, antibiotic use, and other factors in early childhood. We can say that our relationship with the gut microbiota is like a “Sword of Damocles”, enabling conditions of good health while, at the same time, posing potential harm due to a variation derived from various causes (e.g., eating habits, drug abuse). This increases the risk of immune disorders by affecting the basic homeostasis of health, with general and long-term physical effects. The consequences of such dysbiosis will be even more significant if they manifest early in life—a critical period for the maturation of the immune system and development of immune tolerance—affecting the overall effectiveness of the immune system in fighting infections. When the microbial composition changes, non-resistant organisms capable of repelling potential harmful micro-organisms may become pathogenic themselves. However, this finding needs to be further investigated in order to be properly clinically translated. Therefore, large-scale systematic studies with the possibility of providing clinical correlations are needed. The communication pathways between the various axes of the gut microbiota need to be better understood, especially regarding autoimmune phenomena. The main problem in studying human conditions is that the disease itself can already alter any microbiota, especially the intestinal one, which makes it difficult to clearly understand the origin and mechanisms of related autoimmune diseases. We also know that inter-person variability exists in terms of physiological conditions, and that some interventions modify these conditions. Overall, we hope that study of the microbiota will highlight both therapeutic and diagnostic possibilities (regarding potential biomarkers) for a number of autoimmune diseases in the future.

## Figures and Tables

**Table 1:** Examples of molecular mimicry between micro-organisms and human proteins [100].

Molecular Mimicry	
Protein	Sequences
IE2	P D P L G R P D E D
(Cytomegalovirus)	
HLA-DR	
	V T E L G R P D A E
VP2	
(Poliovirus)	S T T K E S R G T T
Acetylcholine receptor	
	T V I K E S R G T K
E2	
(Human papillomavirus; HPV)	S L H L E S L K D S
Insulin receptor (residue 66)	
	V Y G L E S L K D L
Ε1Β	
(Human adenovirus 1)	L R R G M F R P S Q C N
α-Gliadin	
	L G Q G S F R P S Q Q N
P3	
(Μeasles virus; MV)	L E C I R A L K
Corticotropin	
	L E C I R A C K
Glycoprotein complex	
(Lassa virus)	T K E S L V I I S
Insulin receptor (residue 764)	
	N K E S L V I S E
